# Progressive slip after removal of screw fixation in slipped capital femoral epiphysis: two case reports

**DOI:** 10.1186/1752-1947-6-405

**Published:** 2012-11-26

**Authors:** Yde Engelsma, Paul Morgenstern, Hans A Van Der Sluijs, Melinda M Witbreuk

**Affiliations:** 1 Department of Orthopaedics, Postbus 501, Alkmaar, AM, 1800, The Netherlands; 2 Department of Orthopaedics, de Boelelaan 1117, Amsterdam, HV, 1081, The Netherlands

## Abstract

**Introduction:**

In slipped capital femoral epiphysis the femoral neck displaces relative to the head due to weakening of the epiphysis. Early recognition and adequate surgical fixation is essential for a good functional outcome. The fixation should be secured until the closure of the epiphysis to prevent further slippage. A slipped capital femoral epiphysis should not be confused with a femoral neck fracture.

**Case presentation:**

Case 1 concerns a 15-year-old boy with an adequate initial screw fixation of his slipped capital femoral epiphysis. Unfortunately, it was thought that the epiphysis had healed and the screw was removed after 11 weeks. This caused new instability with a progressive slip of the femoral epiphysis and subsequently re-fixation and a subtrochanteric correction osteotomy was obligatory. Case 2 concerns a 13-year-old girl with persistent hip pain after screw fixation for slipped capital femoral epiphysis. The screw was removed as lysis was seen around the screw on the hip X-ray. This operation created a new unstable situation and the slip progressed resulting in poor hip function. A correction osteotomy with re-screw fixation was performed with a good functional result.

**Conclusion:**

A slipped epiphysis of the hip is not considered ‘healed’ after a few months. Given the risk of progression of the slip the fixation material cannot be removed before closure of the growth plate.

## Introduction

Slipped capital femoral epiphysis (SCFE) is the most common adolescent hip disorder. In this condition the metaphysis of the femoral neck displaces anteriorly and superiorly to the femoral head [1]. The epiphysis weakens and eventually fails due to a combination of biomechanical and biological factors [1,2]. This is in contrast to the rare adolescent hip fractures caused by high-energy trauma [3,4]. For a good functional outcome, early recognition and adequate surgical treatment is essential in both cases. In some cases of SCFE the surgical principles of fracture treatment with hardware removal are still used which can lead to a poor outcome [2]. We present two cases with complications after screw removal to highlight the serious consequences of the loss of adequate fixation before the end of growth plate closure.

## Case presentation

### Patient 1

**Figure 1:**
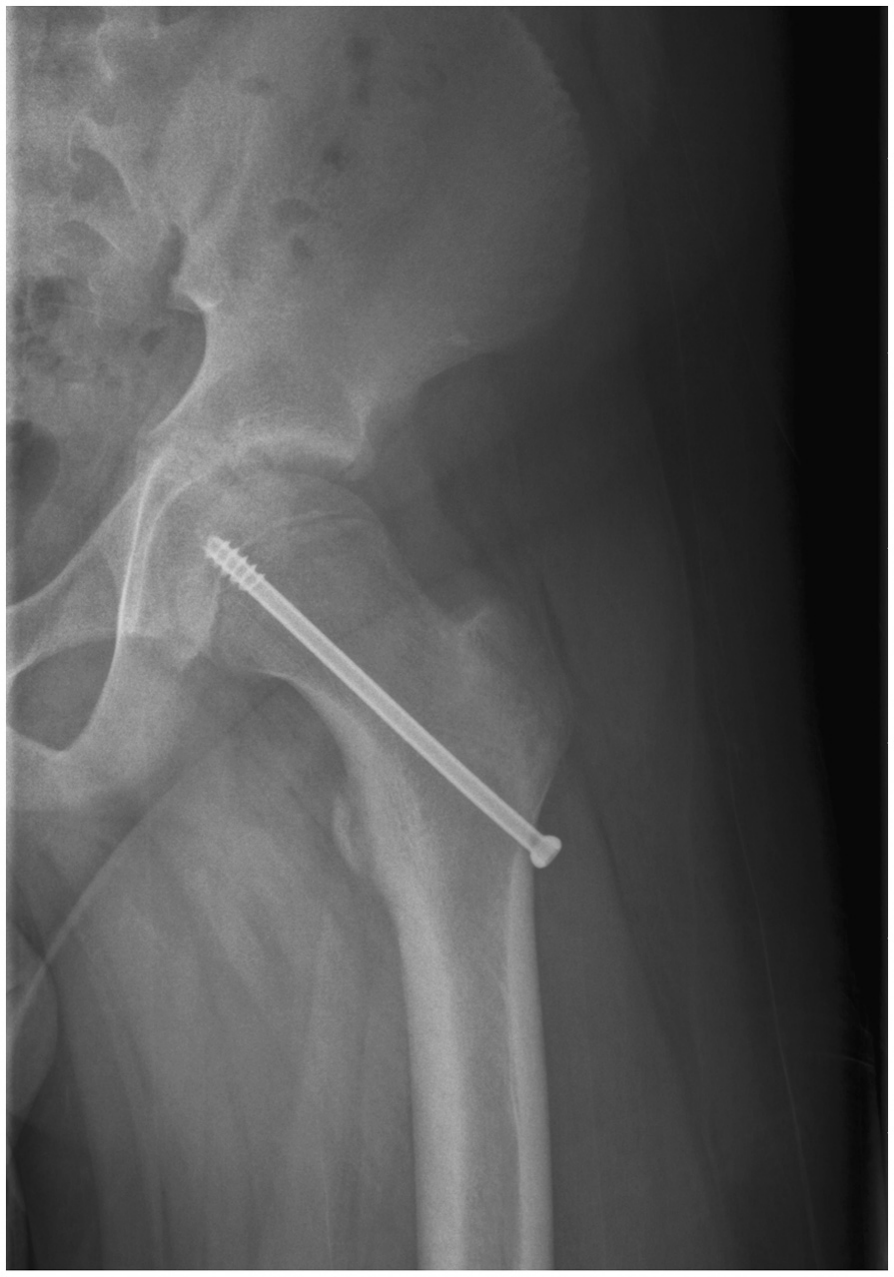
**Patient 1.** The X-ray of the left hip after *in situ* fixation.

A 15-year-old obese boy visited our clinic with a painful hip on the left side. The complaints arose one year earlier after he had fallen on his hip. At that time a mild epiphysiolysis was diagnosed on presentation in the emergency room. He was admitted with bed rest and three days later an *in situ* fixation was performed with one cannulated screw (Figure 1). The postoperative recovery was without complications and he was pain free after a few weeks. As it was thought that the fracture had consolidated, the screw was removed 11 weeks after initial placement. After this procedure had been performed his hip became increasingly painful and he experienced reduced mobility. Ten weeks after the screw removal, he fell again, complaining once again of severe pain in his hip. Plain radiographs were performed and a progressive abnormal position of the head of the hip with callus formation was seen. Initially the conservative treatment consisted of physiotherapy. Because of the persistence of disability he was referred to our orthopedic children’s clinic several months later.

**Figure 2:**
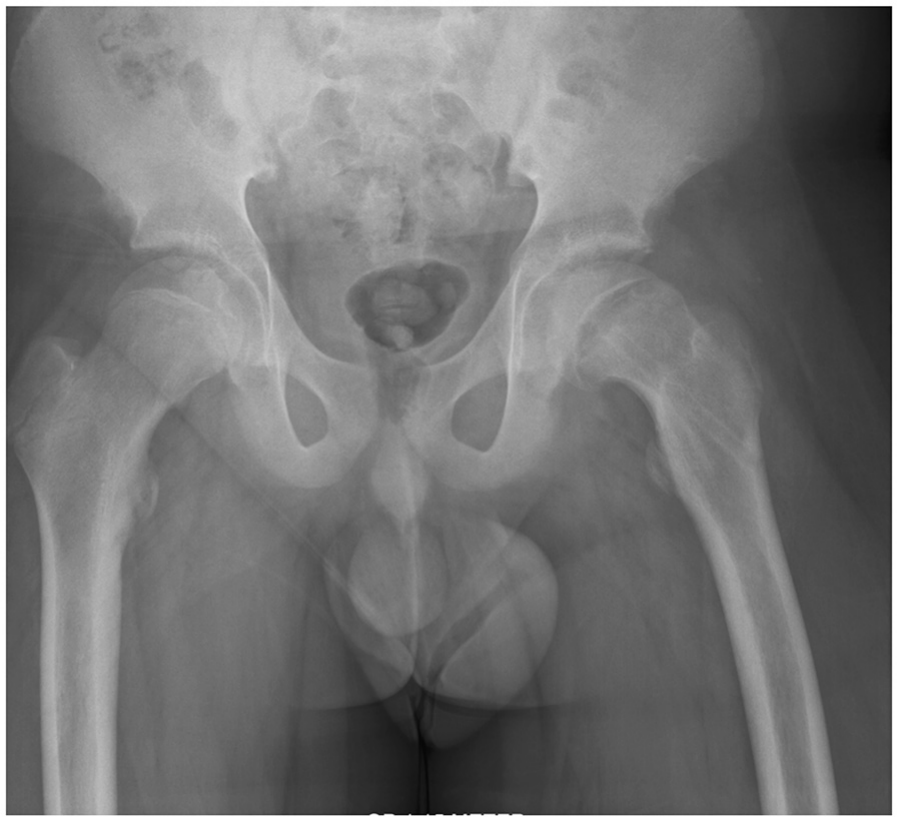
**Patient 1.** The anteroposterior X-ray after removal of the screw fixation shows progression of the slip to nearly 70 degrees.

**Figure 3:**
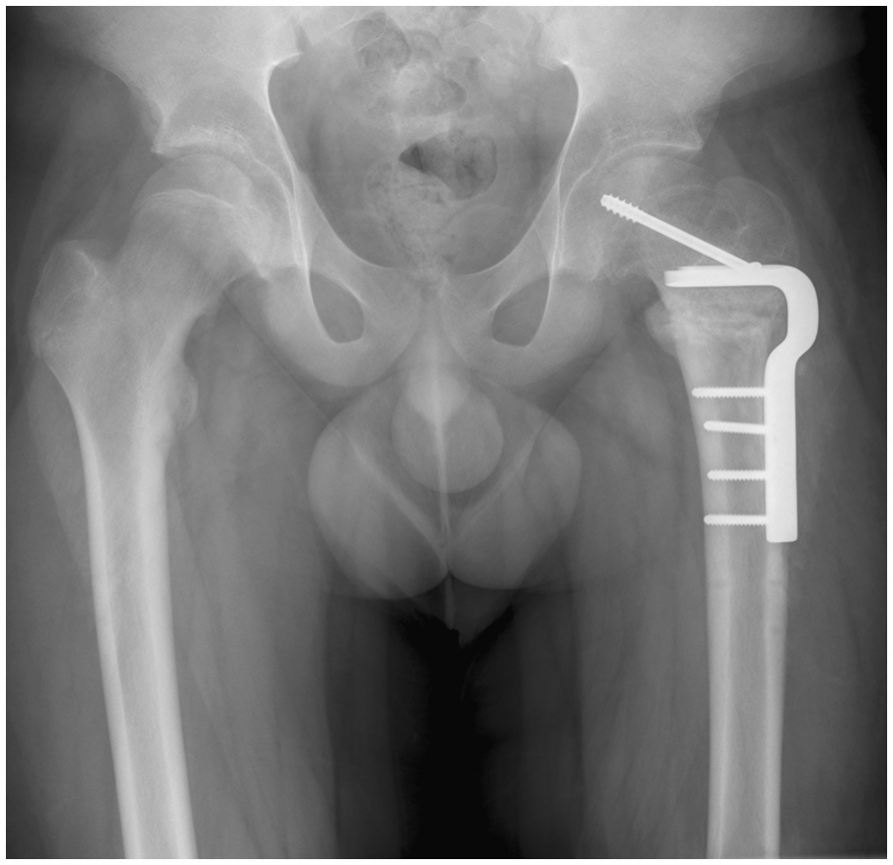
**Patient 1.** The anteroposterior X-ray after the Southwick correction osteotomy with screw fixation of the head.

At that time he had a painful gait with a severely limited left hip function with 70 degrees of flexion. His left leg was externally rotated, with an internal and external rotation in extension of 0–30–50 degrees. The X-rays depicted a severe SCFE with a slip of 70 degrees and an open growth plate (Figure 2). Given the seriousness of the slip and the open growth plate, a re-(screw) fixation of the epiphysis was performed with an additional subtrochanteric correction osteotomy (according to Southwick). The postoperative course was uncomplicated (Figure 3). After an initial period of six weeks of unloaded mobilization, weight bearing was supervised by the physiotherapist. During the last outpatient appointment, two years postoperatively, he was still found to be limping slightly, but he was pain free. On examination there was a leg length difference of 2cm with a hip motion of 100 degrees of flexion and an internal and external rotation of 25–0–45 degrees. The Harris Hip Score was 97. The X-ray showed a cam lesion due to the deformity, no signs of avascular necrosis (AVN) or chondrolysis and a Southwick angle of 25 degrees.

### Patient 2

A 13-year-old girl was referred by her orthopedic surgeon to our orthopedic children’s clinic. A year earlier she had suffered from pain in her left hip and knee after an injury whilst doing gymnastics. The general practitioner had requested only an anteroposterior radiograph of the pelvis, on which no abnormalities were seen. She was started on physical therapy, but pain nevertheless persisted. After referral to an orthopedic surgeon, a mild SCFE was diagnosed on the X-frog-lateral view. She was admitted and on the same day an *in situ* screw fixation was performed. The postoperative course was without problems, but once again her hip remained painful. X-rays depicted a good position of the femoral head, however, there was radiolucency around the screw. It was thought that the persisting pain might be explained by loosening of the screw and it was removed after four months.

The clinical course deteriorated after this procedure and she was referred to our clinic. She had a limping gait and a leg length discrepancy of 1cm. Flexion was limited to 100 degrees on functional assessment of the left hip, and internal and external rotation in extension was 20–0–45 degrees. X-rays demonstrated a moderate SCFE with an open growth plate. A progression of the slip to 50 degrees was present. We decided to perform a correction osteotomy according to Southwick with a re-screw fixation with one screw. Once again there was an initial period of six weeks of non-weight bearing. Her recovery was excellent, and after three months she was able to participate in gymnastics again. At final follow-up at 18 months postoperatively she was able to compete in sports and was almost pain free. On functional assessment, range of motion of the hip was unrestricted, with an internal and external rotation of 45–0–40 degrees. The Harris Hip Score was 96 and the X-ray showed a Southwick angle of 20 degrees and no signs of AVN or chondrolysis.

## Discussion

SCFE is the most common hip disorder in adolescents with an incidence from one to seven per 100,000 [1]. The symptoms can range from a painful gait with minimal restrictions to a very painful condition with a non-weight bearing and an externally rotated leg. It is often (incorrectly) related to a minimal trauma. In case of preceding trauma however, it is essential to differentiate between SCFE and a transepiphysial fracture of the femoral neck. In SCFE the epiphysis becomes weaker and eventually fails due to a combination of biomechanical and biological factors. A genetic component might possibly play a role [1,2]. Hip fractures, by contrast, are extremely rare in children or adolescents and are almost without exception always due to a high-energy trauma [3,4]. In these cases there is no pre-existing biological or mechanical weakening of the growth plate.

A radiological differentiation between the two entities can be difficult. For a proper radiological assessment a frog leg or lateral hip view is essential. In chronic or sub-acute SCFE, remodeling of the bone around the neck is often visible. However, an acute SCFE may have a radiological appearance similar to a transepiphysial fracture [5,6]. The long-term prognosis of SCFE is related to the severity of the initial slip. This is assessed by the method of Southwick on the frog leg or lateral radiograph [1,7]. In the case of a fracture, the Delbet Classification based on fracture location is used [5].

The main goal in the treatment of SCFE is to prevent further slippage and prevent complications such as AVN and chondrolysis. Conservative treatment is not indicated. In a mild to moderate slippage, *in situ* screw fixation is the gold standard [1]. Reduction attempts can cause AVN and are not indicated. However, in an acute or unstable slip, within 24 hours after the onset of symptoms, a tentative reduction attempt can be performed with *in situ* screw fixation [1,2,8]. The addition of a second screw provides only minimal gain in stability with an increased complication risk and is therefore not recommended [1].

If the pain and limping persists after screw fixation one should consider the presence of AVN, intra-articular screw penetration (with chondrolysis) or instability of the growth plate with progression of the slip. If problems arise from the screw fixation itself, it needs to be changed or reversed and not removed. In case of progression of the slip due to instability of the epiphysis a second screw can be added. The fixation must be secured until the growth plate closes to prevent further progression of the disease. Whether or not the material is subsequently removed remains controversial [9]. In the two presented cases the screw was removed with the idea that after a few months the epiphysis had achieved enough stability. However, with an open growth plate the underlying weakness is still present. Removal of the screw therefore causes further progression of the slip and deformity. In both cases, it had considerable consequences for the patient as an additional and more invasive procedure was required.

Treatment of an adolescent transepiphysial hip fracture consists of a closed or open reduction and screw fixation similar to that in SCFE. Removal of the osteosynthesis material after consolidation of these fractures has never been reported and in case of any doubt it is advisable to leave the screw in place [3,4].

## Conclusion

Early diagnosis and timely, adequate surgical stabilization is essential for a good outcome in SCFE. *In situ* fixation with one screw is standard treatment. The growth plate does not heal within several months and the original unstable situation persists until the growth plate is closed. Given the risk of progression of the slip, the fixation of the slipped epiphysis of the hip can only be removed after closure of the growth plate.

## Consent

Written informed consent was obtained from the patients’ legal guardians for publication of this case report and accompanying images. Copies of the written consents are available for review by the Editor-in-Chief of this journal.

## Competing interests

The authors declare that they have no competing interests.

## Authors’ contributions

YE and MW constructed the idea for this study. MW and HS operated on both patients. YE and PM were responsible for the data collection and drafted the manuscript. All authors read, edited and approved the final manuscript.
